# Development of Advanced Materials and Technology for Green and Sustainable Environmental Remediation

**DOI:** 10.3390/ma19101961

**Published:** 2026-05-10

**Authors:** Xing Li, Hao Qiu, Erkai He

**Affiliations:** 1 School of Resources and Environment, Nanchang University, Nanchang 330031, China; lixing1192@ncu.edu.cn; 2 State Key Laboratory of Green Papermaking and Resource Recycling, School of Environmental Science and Engineering, Shanghai Jiao Tong University, Shanghai 200240, China; haoqiu@sjtu.edu.cn; 3 School of Geographic Sciences, East China Normal University, Shanghai 200241, China

With the rapid pace of industrialization and urbanization, environmental pollution has emerged as a critical global challenge that severely threatens ecological health [1]. The escalating detection of traditional pollutants, such as heavy metals and persistent organic pollutants, alongside emerging pollutants, like engineered nanomaterials and microplastics, across environmental matrices underscores the urgency and complexity of strengthening modern environmental governance [2,3,4]. Although conventional environmental remediation technologies have demonstrated measurable efficacy in pollution mitigation, their large-scale engineering application is often constrained by secondary contamination, prohibitive economic expense, and low societal acceptance [5]. Therefore, developing eco-compatible and cost-effective advanced remediation materials is essential for propelling environmental governance into a green, low-carbon, and sustainable future, which has emerged as a core frontier in contemporary environmental science and engineering [6,7]. This Special Issue, entitled “Development of Advanced Materials and Technology for Green and Sustainable Environmental Remediation”, represents recent developments in green remediation materials and their synergistic physicochemical and biotechnological mechanisms in environmental remediation. This Editorial summarizes the key findings of the seven contributions published in this Special Issue, which encompass five original research articles and two comprehensive reviews.

Heavy metals pose a persistent challenge in environmental remediation due to their non-biodegradability and high toxicity [8]. Developing high-capacity adsorbents and utilizing economical by-products for in situ immobilization are essential pathways to effectively mitigate their bioavailability [9]. Naseer et al. synthesized calcite-type calcium carbonate (CaCO_3_) microcubes via a combined co-precipitation and hydrothermal approach [10]. This novel material exhibits an ultra-high Pb^2+^ adsorption capacity of 4018 mg/g through rapid ion-exchange mechanisms, highlighting the material’s viability for efficient heavy metal remediation in aquatic environments. Barseghyan et al. isolated a wild yeast strain (*Rhodotorula* sp.) from acid mine drainages (AMDs) [11]. This strain enables highly efficient, cost-effective, and eco-friendly biosorption of Cu^2+^ and Zn^2+^ under mildly acidic conditions, presenting a promising novel biomaterial for AMD-contaminated water. Rolka et al. explored the application potential of woody biomass ash (BA), a cost-effective combustion by-product, for soil heavy metal remediation, demonstrating that BA can be a highly feasible candidate for sustainable and cost-efficient zinc immobilization [12].

The inadequate disposal of industrial solid waste constitutes a primary source of heavy metal contamination in soil–groundwater systems [13]. Shifting the strategic focus from traditional end-of-pipe treatments to mild-condition resource valorization is pivotal for mitigating environmental risks and advancing the circular economy [14]. Świerczek et al. presented an organic acid-based extraction method for chromium recovery from chromium-tanned leather waste (CTLW), showing that oxalic acid facilitates efficient chromium extraction while preserving collagen integrity and averting the generation of highly toxic Cr(VI) [15]. This method provides a green and viable solution for Cr recovery from CTLW, effectively filling a crucial gap in industrial solid waste treatment.

Accidental oil spills and the subsurface seepage of organic contaminants pose severe threats to the ecological security of soil–groundwater systems [16]. Constructing engineered contaminant containment systems is crucial for shifting geo-environmental management from passive restoration to active risk containment [17]. Lee et al. developed an advanced hybrid liner system that combines physical barriers, reactive barriers, and absorptive systems to rapidly immobilize total petroleum hydrocarbons in situ, thereby preventing their vertical migration without perturbing groundwater hydrodynamics [18].

Beyond conventional chemical contaminants, emerging biological and particulate pollutants such as pathogenic microorganisms and microplastics are garnering unprecedented attention, owing to their pronounced environmental persistence and pervasive mobility [19,20]. Hrenović and Rajić summarized the antibacterial properties of natural zeolites modified by transition metals and metal-oxide nanoparticles, highlighting the broad application landscape of natural zeolites for sustainable and eco-friendly disinfection [21]. Xiao et al. systematically reviewed recent advances in the application of nanomaterials for the photodegradation of microplastics. By emphasizing photocatalyst optimization strategies (element doping and heterojunction construction), their research offers novel insights and technological pathways toward the complete mineralization of microplastics [22].

Currently, the development of environmental functional materials has reached a critical crossroads, evolving from the reduction in conventional pollutants toward precision-driven, scenario-specific, and full-life-cycle sustainable paradigms [23,24,25]. To effectively bridge the gap between laboratory research and the full-scale engineering application of green remediation technologies, it is imperative to develop eco-compatible functional materials from the perspectives of material design, safety effects, and full Life Cycle Assessment (LCA) in the future. Firstly, the next-generation functional materials must overcome the monofunctional limitations of traditional counterparts, pivoting to address the formidable challenges of complex environmental media and elusive emerging contaminants [26,27,28]. Secondly, future remediation strategies should aim not only to eliminate target contaminants, but also to mitigate the potential environmental risks induced by the applied materials and the transformation of pollutants [29,30,31,32]. Lastly, the large-scale application of advanced environmental functional materials must be rigorously incorporated into an LCA framework, ensuring the synergistic achievement of optimal decontamination performance and low-carbon sustainable objectives [33,34,35].
